# Neurogenesis, Exercise, and Cognitive Late Effects of Pediatric Radiotherapy

**DOI:** 10.1155/2013/698528

**Published:** 2013-04-14

**Authors:** Shaefali P. Rodgers, Melissa Trevino, Janice A. Zawaski, M. Waleed Gaber, J. Leigh Leasure

**Affiliations:** ^1^Department of Psychology, University of Houston, Houston, TX 77204, USA; ^2^Department of Pediatrics, Texas Children's Hospital, Baylor College of Medicine, Houston, TX 77030, USA; ^3^Department of Biology & Biochemistry, University of Houston, Houston, TX 77204, USA

## Abstract

Brain cancer is a common type of childhood malignancy, and radiotherapy (RT) is a mainstay of treatment. RT is effective for tumor eradication, and survival rates are high. However, RT damages the brain and disrupts ongoing developmental processes, resulting in debilitating cognitive “late” effects that may take years to fully manifest. These late effects likely derive from a long-term decrement in cell proliferation, combined with a neural environment that is hostile to plasticity, both of which are induced by RT. Long-term suppression of cell proliferation deprives the brain of the raw materials needed for optimum cognitive performance (such as new neurons in the hippocampus and new glia in frontal cortex), while chronic inflammation and dearth of trophic substances (such as growth hormone) limit neuroplastic potential in existing circuitry. Potential treatments for cognitive late effects should address both of these conditions. Exercise represents one such potential treatment, since it has the capacity to enhance cell proliferation, as well as to promote a neural milieu permissive for plasticity. Here, we review the evidence that cognitive late effects can be traced to RT-induced suppression of cell proliferation and hostile environmental conditions, as well as emerging evidence that exercise may be effective as an independent or adjuvant therapy.

## 1. Introduction

Brain tumors are the second most common form of childhood cancer, after acute lymphoblastic leukemia (ALL) [1]. Treatment for both brain tumors and ALL includes cranial RT. Given 5-year survival rates that approach 90% for children treated for ALL and 70% for those treated for brain tumors [2], there are currently a great many survivors of these cancers that suffer from the consequences of RT, including adverse physiological, psychological, and cognitive side effects that manifest both acutely and years later. These so called “late effects” result in lowered quality of life (QOL) [3] in survivors, for which there is at present no effective treatment.

RT for pediatric cancer has long been acknowledged as a primary cause of neurological complications and neurocognitive decline [4–8]. Childhood RT is associated with a significant decrease in IQ scores [8–14], thought to result from deficits in core processing functions impaired by RT, including processing speed [15], attention [15–18], working memory, and other executive functions [7, 19]. In addition to cognitive impairments, adult survivors of childhood RT also experience elevated rates of emotional distress, such as anxiety and/or depression [20, 21] and posttraumatic stress disorder [22]. These cognitive and emotional consequences of RT result in decreased QOL that manifests in a variety of ways. For example, adult survivors of childhood RT are less likely to obtain a college education [23, 24] or marry [5, 20] and more likely to be unemployed [24, 25].

Improving QOL for survivors necessarily involves attenuating the long-term neural consequences of RT. Ionizing radiation damages the brain directly, but in addition, it chronically suppresses cell proliferation, thereby depriving the brain of the raw materials needed for repair. Evidence indicates that it also creates a milieu that is hostile to regenerative processes. When the brain is irradiated in childhood, there is a further consequence of RT, as suppressed cell proliferation and hostile environmental conditions disrupt ongoing developmental processes. What is needed, therefore, is a treatment that can both “jump-start” cell proliferation and foster a neural environment that is conducive to plasticity. Exercise may represent one such treatment, and its restorative potential for the post-RT brain is discussed.

## 2. RT Disrupts Brain Development

RT damages the brain regardless of age. However, the brains of children are still developing, and RT profoundly affects ongoing developmental processes. The potential mechanisms underlying this disruption are many, such as perturbations of vasculature [26] and suppression of cell proliferation [27–29]. Damage to the endocrine system [30, 31] has been shown to play a role, in particular, decreased expression of growth hormone (GH). GH deficiency results from the effects of a brain tumor or of therapy such as surgery, RT, or chemotherapy. Merchant et al. [32] report that the peak GH response within 12 months after the initiation of cranial RT depends on hypothalamic dose-volume effects and may be predicted on the basis of a linear model that sums the effects of the entire dose distribution. The rate of decline in the peak GH response may also be influenced by clinical factors indicating the severity of the disease and the type and location of tumor.

Disruption of brain development could also be due in part to cancer treatment effects on food intake. Treatment-induced nausea and vomiting, as well as gastrointestinal toxicity can lead to nutritional deficiency and changes in body composition [34, 35], which may be long-lasting. Indeed, survivors of childhood brain cancer are often underweight [36]. In contrast, survivors of childhood ALL are more likely to be obese, compared with age-matched controls [37]. Thus, treatment effects on hormone levels and nutritional intake, alone or in combination, are likely important contributors to altered neural development and, ultimately, cognitive impairments.

**Figure 1:**
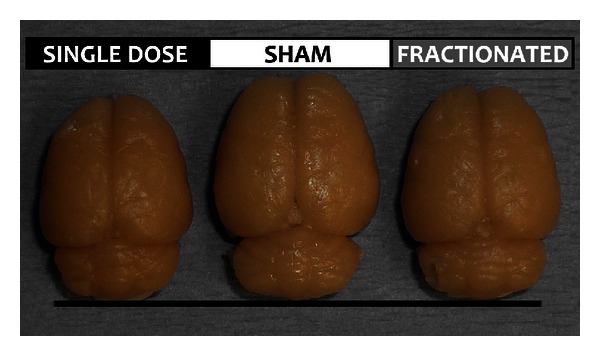
Irradiation of the developing (PND28) rat brain results in visibly decreased brain size in adulthood. Note that a 20 Gy total dose of X-ray radiation resulted in a smaller brain when it was administered as a single dose. A fractionated dose (4 Gy/d for 5 days) was less detrimental to brain size.

Animal models of pediatric RT enable controlled study of mechanisms that contribute to disrupted development and, ultimately, cognitive late effects. To model the effects of RT on the developing brain, we have treated postnatal day 28 (PND28) rats with whole brain irradiation (WBI), using one of 2 regimens: single dose (20 Gy) or fractionated, in which animals received 20 Gy over the course of 5 days (4 Gy/d). Either regimen results in a profound stunting of brain growth visible to the naked eye (see Figure 1), although the effect is clearly bigger with single dose treatment.

**Figure 2:**
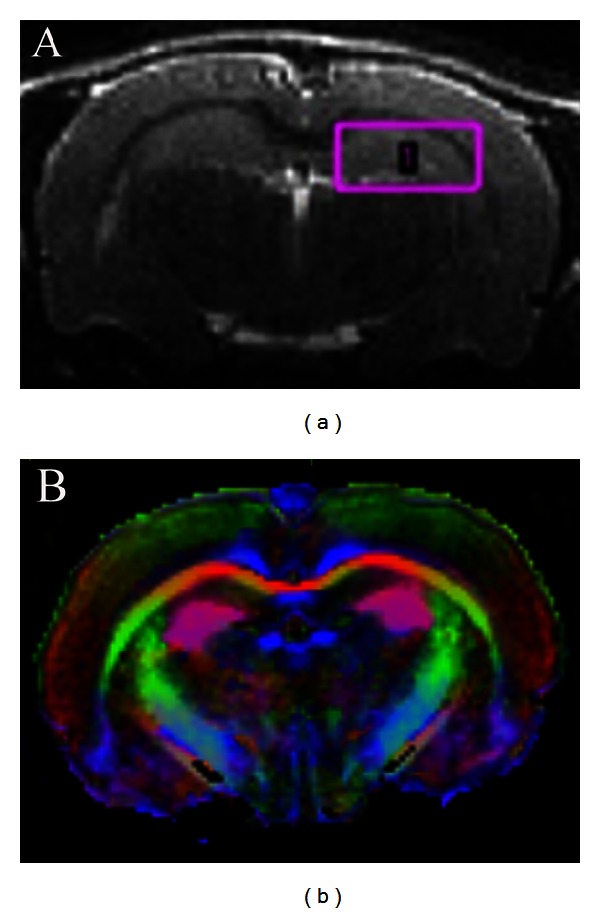
Data from our group indicates that measurable imaging changes precede cognitive decline. (a) An image of a rat brain acquired using a 9.4 T MRI. The pink box indicates where ^1^H MRS was performed. Changes in glutamate, alanine, and lactate preceded cognitive impairments. In addition, FA analysis detected a decrease in volume of the fimbria. (b) An FA map of the rat brain.

**Figure 3:**
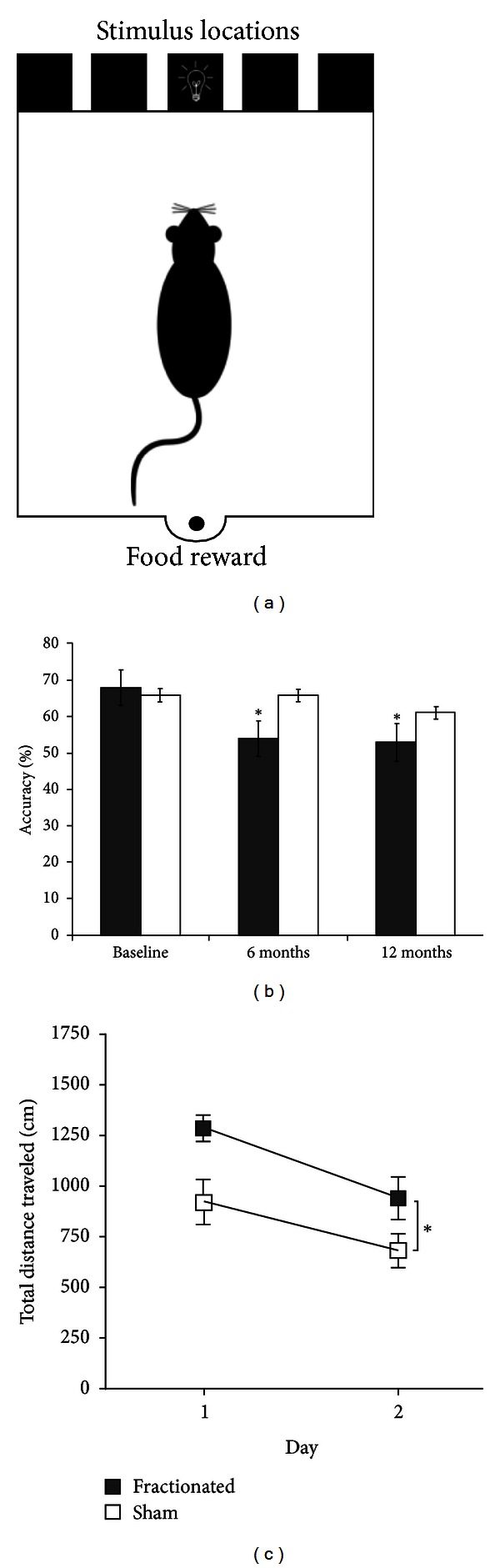
Data from our group showing RT-induced cognitive deficits. (a) Schematic illustration of the 5-CSRTT apparatus. (b) Fractionated X-ray radiation (4 Gy/d for 5 days) restricted to the frontal cortex of young rats significantly reduced choice accuracy on a 5-CSRTT 6 and 9 months following RT (*P < 0.05). (c) Fractionated WBI in young rats impaired pliancy on a hippocampus-dependent strategy-switching task in the Morris water maze [33] 3-4 weeks following RT (*P < 0.05).

Using this model, we can probe the cellular, chemical, and structural effects of RT that contribute to decreased brain size and cognitive impairments in adulthood. To enhance translational value, we are using imaging techniques to discover RT-induced changes *in vivo* that predict future cognitive impairments before they manifest. For example, we are using magnetic resonance imaging (MRI) and diffusion tensor imaging (DTI) to assess RT-induced structural changes and ^1^H magnetic resonance spectroscopy (MRS) to assess chemical changes following RT (see Figure 2). DTI has the added advantage of providing information on fractional anisotropy (FA), a measure of the functional integrity of white matter tracts. Our preliminary ^1^H MRS findings showed changes in glutamate, alanine, and lactate in RT brains, compared to sham controls. In addition, FA analysis showed a significant decline in fimbria volume and mean fimbria FA value in RT brains compared to controls. These changes were observed three months prior to the detected cognitive changes shown in Figure 3, suggesting that imaging changes can be used as early markers of cognitive decline.

## 3. RT-Induced Suppression of Cell Proliferation Contributes to Cognitive Impairments

Because ionizing radiation kills dividing cells, it is effective at treating cancer, yet devastating to noncancerous tissue in the brain. Although mature neurons are postmitotic and therefore not directly affected by radiation, the brain's actively dividing neural stem cells (NSC) are largely wiped out, even by very low doses [28]. This is problematic, as it decreases the availability of new neurons in neurogenic regions of the brain and of new glia (oligodendrocytes and astrocytes) in nonneurogenic areas.

The dentate gyrus (DG) of the hippocampus, along with the lining of the lateral ventricles (the subventricular zone, or SVZ), is one of the few neurogenic regions of the adult mammalian brain (see [38] for review). Animal studies indicate that ongoing neurogenesis in this region is important for cognition. For example, newly generated neurons are kept alive by effortful learning (for review see [39]) and are needed for the formation of long-term spatial memory [40]. Analysis of postmortem human tissue following cancer treatment shows an almost complete lack of hippocampal neurogenesis [41], the functional importance of which is attested to by the cognitive impairments observed in survivors [42].

Animal models have yielded direct links between RT-induced decrements in hippocampal neurogenesis and cognitive impairments. Many studies have focused on deficits in spatial performance (place learning or spatial memory) and trace fear conditioning, since these are hippocampus-dependent functions. Spatial impairments have been observed in conjunction with decrements in DG cytogenesis following both fractionated [43, 44] and single-dose WBI [45, 46]. Our group also has noted performance deficits in a spatial task after fractionated irradiation (see Figure 3(c)). Decreased fear conditioning has also been associated with radiation-induced suppression of DG cytogenesis [47–49]. In sum, an increasing body of evidence implicates suppression of hippocampal neurogenesis as a causative factor in cognitive impairments following RT.

However, suppression of hippocampal neurogenesis is likely only part of the story. NSCs in nonneurogenic brain regions, such as the cortex, differentiate into glia [50]. A plentiful supply of glial cells is essential for neuronal health and function [51, 52], so reduced proliferation of NSCs due to RT could contribute to cognitive impairments by reducing the availability of glia. For example, problems with executive functions are widely reported in adult survivors of childhood RT. Executive functions develop linearly during adolescence, in apparent conjunction with myelination of the frontal lobes [19]. Frontal lobe white matter appears particularly vulnerable to RT [53], and RT-induced damage to white matter tracts may, in large part, underlie the neurocognitive deficits experienced by adult survivors of childhood cancer [19, 54]. Myelination is dependent on a ready supply of healthy oligodendrocytes, which is in turn dependent on adequate proliferative activity of NSCs. RT-induced ablation of NSCs in nonneurogenic regions could therefore contribute to cognitive impairments. 

To provide direct evidence that RT-induced suppression of gliogenesis contributes to frontal lobe dysfunction, animal models of frontal lobe-dependent tasks are important. The 5-choice serial reaction time task (5-CSRTT) is a reliable means by which to assess prefrontal cognitive processes in the rodent. This automated task measures several aspects of visual attention, specifically divided, sustained, and selective attention, as well as processing speed and impulsivity [55]. The task requires the animal to detect brief flashes of light that appear in one of five apertures (see Figure 3(a)) and then nose-poke into the aperture that the light appeared in. The animal is given 5 seconds in which to make the nose-poke response. Correct responses are rewarded by a food pellet being dispensed into a magazine at the rear of the testing chamber (see Figure 3(a)). To provide motivation, animals are food restricted. Between stimulus presentations, there is an intertrial interval (ITI), and the animal must inhibit responding during this interval, because premature responses result in a short time-out period during which there are no trials, and thus food reward cannot be obtained. In performing this task, the animal has to sustain attention to all 5 of the apertures in order to constantly monitor where the light stimulus will be presented. Incorrect responses (nose-pokes into an aperture other than that in which the light was presented) indicate impaired attention. Measures of impulsivity are collected through responses that are characterized as perseverative and/or premature. Perseverative responses are defined as continuous nose-pokes in additional apertures. Nose-pokes made before the light is presented are considered premature responses. Processing speed measures are based on various latency times that are collected throughout the task.

We have used the 5-CSRTT to probe for impairment of prefrontal cognitive processes following fractionated irradiation (4 Gy/d for 5 days). Irradiated animals and shams were trained to perform the 5-CSRTT and then tested 6 and 9 months postirradiation. Our preliminary findings indicate that the irradiated animals are significantly less accurate at nose-poking into the correct aperture, suggesting that they have attentional impairments (see Figure 3(b)). Future experiments will focus on replicating these impairments in irradiated animals and determine whether they are linked to reduced gliogenesis in frontal regions.

## 4. RT Creates a Brain Milieu Hostile to Plasticity

**Figure 4:**
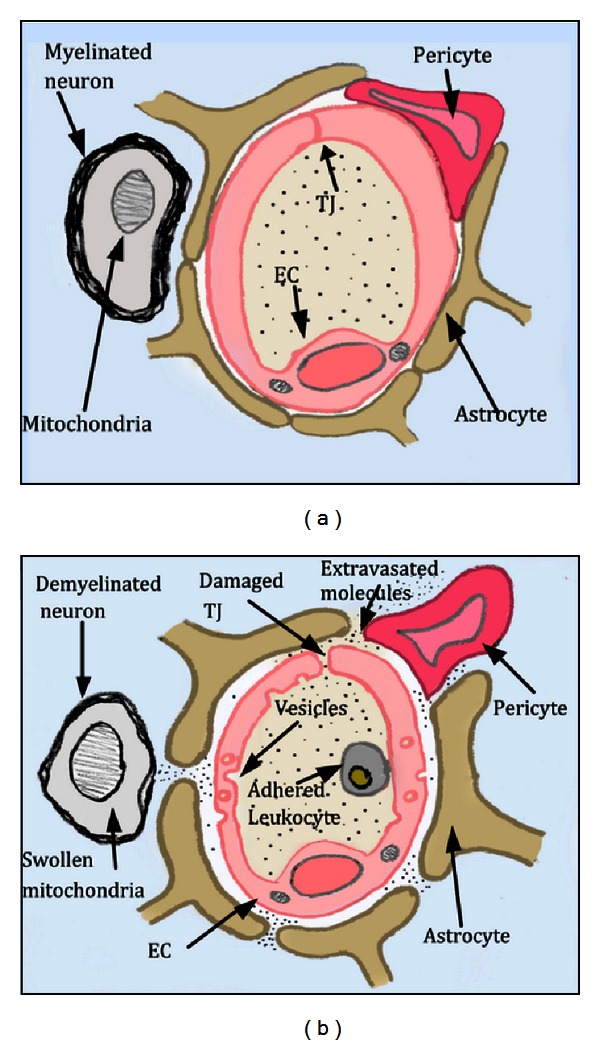
This diagram depicts a cross section of brain parenchyma showing the structure of the BBB and the damage induced by RT. (a) Normal BBB showing intact tight junctions (TJ), lack of vesicles, astrocytes and pericytes abutting the EC providing additional barrier support, and a neuron with thick, healthy myelin. (b) Damaged BBB in which astrocytes and pericytes have pulled away from the EC, a leukocyte has adhered to the EC, and there is formation of vesicles and loss of TJ integrity.

The microenvironment of the brain is regulated and protected by specific barriers, which include the vascular endothelial barrier (also called the blood-brain barrier, or BBB) at the capillary-parenchyma interface and the epithelial barrier (blood-cerebrospinal fluid barrier) at the choroid plexus [56]. The BBB is more than a physical barrier: it plays a fundamental role in regulating the movement of substances between the blood and the CNS (see Figure 4(a)). The microvascular network is also the site of the BBB, and the endothelial cells (ECs) that make up the microvascular network barrier contain few pinocytotic vesicles and adhere to each other via tight junctions [57]. Tight junctions limit paracellular transport of hydrophilic compounds into the CNS as compared to non-CNS vessels [58, 59]. Also, astrocytes in close proximity to the ECs add another impediment to paracellular transport by biochemically conditioning the ECs and strengthening the tight junctions between them [60]. ECs coat, in a single layer, the interior of all blood vessels. Because of this intertwined fate with the circulatory system, ECs play a unique role in maintaining physiological homeostasis, controlling the movement of substances across from the blood compartment into the different tissues and organs with varying demands and function [61]. The ECs also play an important immune function through leukocyte surveillance and extravasation by regulating adhesion integrins and cytokine production [62]. In particular, they have been shown to directly secrete tumor necrosis factor (TNF) [63]. Thus, damage to the ECs compromises the integrity of the BBB.

When the barrier between the vascular supply of the brain and the CNS parenchyma is disrupted, excess extravasation of proteins, biologic-response molecules (e.g., growth factors, cytokines, and clotting factors), inflammatory cells, and therapeutic drugs can damage the brain [56, 64–66]. The disruption of the BBB (see Figure 4(b)) has been identified as a consequence of various diseases/injuries such as multiple sclerosis, ischemia, HIV, hypertension, brain tumors, CNS injury, and radiation exposure [66–70], wherein inflammatory cells are able to penetrate the BBB and destroy the myelin surrounding the axons. Demyelination and myelin thinning have been reported in the CNS following RT [71–74]. Felts et al. have also shown that RT-induced BBB permeability prolonged the induced demyelination of neurons [75, 76]. We and others [77–80] have demonstrated that there is an increase in BBB permeability following RT, which is caused in part by EC damage, as expressed by changes in tight junction integrity and by vesicle formation postirradiation. RT-induced EC damage has been investigated [81–83] with the aim of elucidating the effect on initiating and/or sustaining radiation side effects. Eissner et al. [84], as well as others [82, 83], have shown that when irradiated, ECs *in vitro* and *in vivo* undergo apoptosis at a higher percentage than other cells. Our studies using electron microscopy show that RT causes damage to the tight junctions [78], which is also connected to the observed increase in BBB permeability. In addition, several studies, including our own, have shown an increase in BBB permeability and an increase in the number of vesicles following fractionated cranial irradiation [78–80].

**Figure 5:**
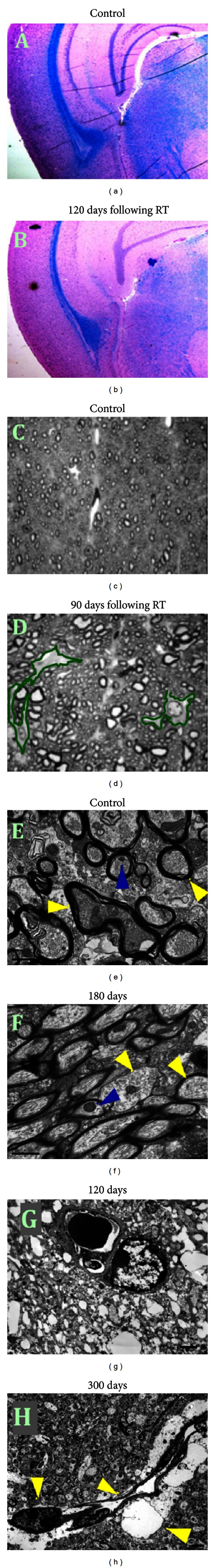
Histological markers of radiation injury in the mouse brain at 90, 120, 180, and 300 days after RT. (a, b) Luxol fast blue staining showing loss of myelin. (c, d) Sections of brain nerve fibers showing structural changes, microglial (outlined in green) inflammation, and myelin sheath thickening indicative of cell death (images at 50x). (e, f) Yellow and blue arrow heads point at myelin sheath surrounding the neurons and at mitochondria, respectively (scale bar = 1 *μ*m). (g) Necrosis detected at 120 days. (h) Yellow arrows point at pericyte pulling away from endothelial cell, a sign of inflammation, with white edematous area causing vascular constriction.

Such damage to the microvasculature and breach of the BBB can disturb the delicate brain microenvironment and expose the brain parenchyma and neural cells to noxious substances [70, 78, 85]. This microenvironment imbalance can set into motion a chain of events (such as cytokines release), magnifying the original signal and finally causing measurable late-term tissue damage in the irradiated brain that may play a role in cognitive impairment [86]. We and others have shown that RT induces an inflammatory response as indicated by an increase in TNF-*α* and intercellular adhesion molecule-1 (ICAM-1) signaling in the brain [87–90]. We have reported activated astrocytes after treatment with single and fractionated RT [78, 91]. Prolonged gliosis can create glial scar sites, which have been theorized to inhibit axonal regeneration or remyelination [92, 93]. We have demonstrated that this inflammation response is related to an increase in BBB permeability following RT and that it is abrogated when treated with antibodies to TNF-*α* or ICAM-1 [77, 90]. In a histological study on mouse brains we observed significant changes 120 days following fractionated RT: fewer neurons, a significant decrease in myelin suggesting complete destruction of the parts of the white matter at 120 days following RT, and at 90 days following RT, we observed swelling of nerve fibers and increased thickening of the myelin sheaths (see Figure 5(b)) indicative of dying axons. 

## 5. The Restorative Potential of Exercise for the Post-RT Brain

Given its myriad beneficial effects on the brain, exercise has been suggested as a treatment for a wide variety of brain maladies, from aging [94] to alcoholism [95]. In the case of aging, exercise has been shown to have a remarkable restorative effect, encouraging the resurgence of atrophied regions such as white matter tracts [96] and the hippocampus [97], and improving cognition [98]. Such effects are particularly encouraging for the post-RT brain, since it shares many things in common with the aged brain, such as decreased cell proliferation, decreased growth hormone, and increased inflammation. Moreover, in both cases these conditions worsen over time, to ultimately create a neural milieu in which plasticity is suppressed.

In aged rodents, exercise can increase proliferation of NSCs [99], suggesting that it has neurogenic potential even in a system in which cell proliferation is drastically reduced. Encouragingly, exercise has been reported to increase hippocampal neurogenesis in the irradiated brain in rodent models [100, 101]. There are likely multiple mechanisms of this enhanced neurogenesis. Neurogenesis is tightly linked to the microenvironment [102] and is known to be suppressed under conditions in which there is unchecked inflammation [27] or a lack of trophic [103] or hormonal support [104]. Exercise has been shown to increase growth hormone [104] and reduce inflammation [105], two potential ways in which it could counteract the suppressive environment created by RT.

**Figure 6:**
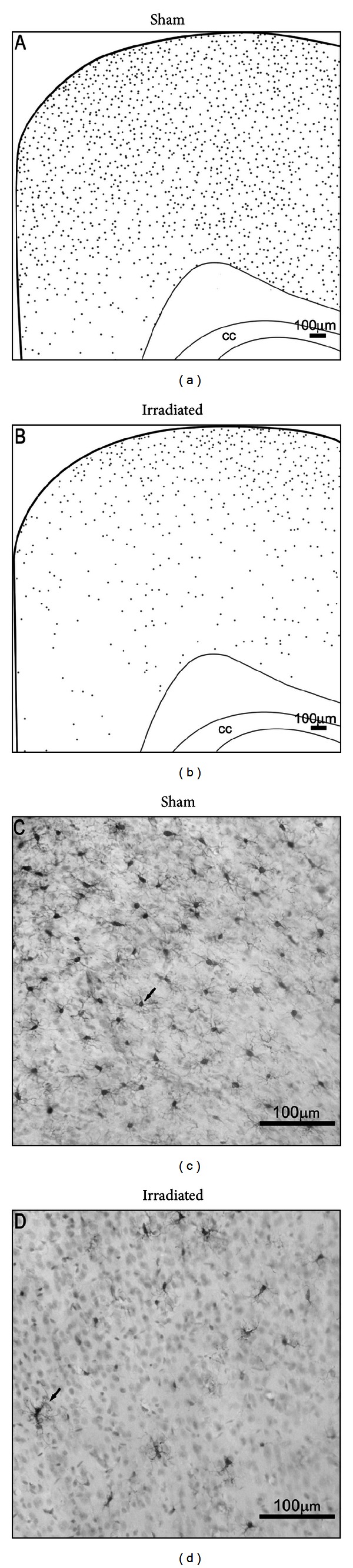
Effect of a single 4 Gy dose of X-ray radiation on microglia in the developing rat brain 24 hr after exposure. (a, b) A schematic representation of microglia distribution (gray dots) in the cerebral cortex showing that RT-induced loss of microglia is more pronounced in inner layers relative to superficial layers. (c, d) Representative 20x images of Iba1+ staining in the retrosplenial cortex showing that RT not only reduces the number of microglia but also alters their morphology.

Recent research has begun to elucidate the important role that microglia have in maintaining the neurogenic niche [106–108]. Unfortunately, radiation severely disrupts microglial distribution, alters their morphology (see Figure 6), and decreases their numbers [109], effects that likely contribute to RT-induced neurogenesis impairment. It has been shown that, after radiation, microglia in the SVZ rebound more quickly than those in the DG. This may explain why neurogenesis recovers better in the SVZ, compared to the dentate [110]. Voluntary exercise has been shown to increase microglia [111], suggesting a further means by which exercise could help to restore a microenvironment conducive to cell proliferation.

Exercise might also have an enhancing effect on gliogenesis in the post-RT brain. As described above, glia are essential for the integrity and function of the cortex. Exercise has been shown to enhance cortical gliogenesis in the intact brain [112], and our future efforts will include determining whether post-RT exercise enhances cortical gliogenesis and ameliorates impairments in the 5-CSRTT.

In addition to these many direct benefits, it is important for survivors of childhood RT to exercise, in order to counteract chronic conditions that arise from cancer treatment, such as impaired pulmonary and cardiac function [113]. Emotional problems like depression and anxiety may also decrease with regular exercise. Unfortunately, treatment-induced fatigue, cardiorespiratory problems, and muscular deconditioning tend to promote sedentary habits during treatment that linger into adulthood, with the result that childhood cancer survivors are much less physically active than their healthy peers (see [113] for review). Cranial RT in particular is associated with sedentary habits in adulthood [114, 115]. However, recent studies suggest that physical fitness is an achievable goal for childhood cancer survivors [36, 113], so the beneficial effects of exercise observed in animal models can be followed up in human patients. 

Fortunately, rodents show no reluctance to exercise after RT, and initial studies suggest that exercise is capable of ameliorating RT-induced deficits in both neurogenesis and cognition. Voluntary running in adulthood has been shown to restore neurogenesis in mice irradiated early in life [100], suggesting that exercise may be a feasible means by which to promote cell proliferation in adult survivors of childhood RT. Furthermore, it may be able to attenuate RT-induced cognitive impairments. A recent study showed that voluntary running ameliorated radiation-induced spatial memory decline 4 months after radiation as well as partially restored neurogenesis in the DG [101].

While these results are encouraging, continued study of the effects of exercise on the RT brain in animal models is essential. For one thing, it is important to better understand the effects of exercise in the context of the RT brain. In particular, it is necessary to determine whether exercise has an adequate neural substrate on which to work. For example, one well-established effect of exercise is its ability to induce angiogenesis [116, 117], an effect that depends upon the brain's capacity to produce new ECs. Given the suppressive effect of RT on cell proliferation, it is possible that the angiogenic effect of exercise would be limited in the post-RT brain. In short, it is possible that the effects of exercise would be dampened in the post-RT brain, reducing its potential as a stand-alone treatment. Further study may indicate that exercise would be most useful as an adjuvant therapy. For example, stem cell replacement shows promise in the irradiated rodent brain [118] and may eventually be possible in humans if the hostile environment created by RT can be made more permissive for growth and repair. Exercise represents a viable means by which to achieve this, and future studies should address the potential of exercise to neutralize the hostile environment created by RT, as a preliminary step in restorative treatments.

## 6. Conclusions

Both human and animal studies indicate that suppressed cell proliferation and the hostile neural environment induced by cranial RT contribute to subsequent cognitive impairments. These effects of RT may be alleviated, at least to some extent, by exercise. It has been well established that exercise can both promote cell proliferation as well as foster a neural environment permissive for plastic change. Early evidence from animal models indicates that exercise has the capacity to do both in the post-RT brain. However, further studies are needed, in order to determine whether RT-induced perturbations of the microenvironment could limit the plasticity-enhancing effects exercise has to offer.
